# Hydrogel-based delivery system applied in the local anti-osteoporotic bone defects

**DOI:** 10.3389/fbioe.2022.1058300

**Published:** 2022-11-11

**Authors:** Yining Gong, Yazhong Bu, Yongliang Li, Dingjun Hao, Baorong He, Lingbo Kong, Wangli Huang, Xiangcheng Gao, Bo Zhang, Zechao Qu, Dong Wang, Liang Yan

**Affiliations:** ^1^ Department of Spine Surgery, Honghui Hospital, Xi’an Jiaotong University, Xi’an, China; ^2^ Department of Biophysics, Institute of Medical Engineering, School of Basic Medical Sciences, Health Science Center, Xi’an Jiaotong University, Xi’an, China; ^3^ Department of Rehabilitation, Honghui Hospital, Xi’an Jiaotong University, Xi’an, China

**Keywords:** hydrogel, delivery system, osteoporosis, osteoclast, osteoblast, local treatment

## Abstract

Osteoporosis is an age-related systemic skeletal disease leading to bone mass loss and microarchitectural deterioration. It affects a large number of patients, thereby economically burdening healthcare systems worldwide. The low bioavailability and complications, associated with systemic drug consumption, limit the efficacy of anti-osteoporosis drugs currently available. Thus, a combination of therapies, including local treatment and systemic intervention, may be more beneficial over a singular pharmacological treatment. Hydrogels are attractive materials as fillers for bone injuries with irregular shapes and as carriers for local therapeutic treatments. They exhibit low cytotoxicity, excellent biocompatibility, and biodegradability, and some with excellent mechanical and swelling properties, and a controlled degradation rate. This review reports the advantages of hydrogels for adjuvants loading, including nature-based, synthetic, and composite hydrogels. In addition, we discuss functional adjuvants loaded with hydrogels, primarily focusing on drugs and cells that inhibit osteoclast and promote osteoblast. Selecting appropriate hydrogels and adjuvants is the key to successful treatment. We hope this review serves as a reference for subsequent research and clinical application of hydrogel-based delivery systems in osteoporosis therapy.

## 1 Introduction

Osteoporosis is an age-related systemic skeletal disease leading to bone mass loss and microarchitectural deterioration, leading to increased bone fragility and susceptibility to fracture (Compston et al., 2019). The World Health Organization, based on standard deviation scores for bone mineral density (BMD), set the criteria to diagnose osteoporosis as a BMD T-score of –2.5 or less (Kanis, 1994). The prevalence of osteoporosis was found to be higher for postmenopausal women (32.1%) compared to men aged 50 years or older (6.9%) (Wang L. et al., 2021). In addition, the onset of vertebral compressive fractures among men and women affected by osteoporosis and aged 80 years or older is nearly 40%, leading to a high rate of mortality, and in turn increased healthcare costs (Kado et al., 2003; Burge et al., 2007; Schousboe, 2016; Wang L. et al., 2021). In fact, nearly six million osteoporosis-related fractures are expected to occur annually by 2050, accounting for an expenditure of over $ 25.43 billion (Si et al., 2015).

Despite the intrinsic self-repairing properties of bone tissue, its regeneration is hindered in the complex osteoporotic pathological environment; therefore, severe bone defects often require targeted treatments promoting bone formation or anti-resorptive therapies (Zhao et al., 2020; Macías et al., 2021). Pharmacological therapy is commonly used for high-risk patients in the absence of contraindications, such as bisphosphonate (BP) drugs (Khosla et al., 2012; McClung et al., 2013), receptor activator of nuclear factor κB ligand (RANKL) inhibitors (Rachner et al., 2011), hormone-replacement (Cauley et al., 2003), selective estrogen-receptor modulators (Ettinger et al., 1999), and parathyroid hormone-related protein (Neer et al., 2001; Miller et al., 2016). Despite anti-resorptive effects and reduced fracture risks, the low bioavailability and the high risk of complications linked to the systemic use of these drugs limit their application (Khan et al., 2015; Tan et al., 2016). For example, the very low bioavailability of alendronate (0.6%) may cause renal dysfunction, hypocalcemia, osteonecrosis of the jaw, and esophageal ulceration after excessive use (Khan et al., 2015; Posadowska et al., 2015; Hosny and Rizg, 2018; Nafee et al., 2018). Thus, the development of a local delivery system assumes a high practical significance in the clinical treatment of osteoporosis.

Hydrogels are cross-linking hydrophilic polymer chains arranged in 3D networks, exhibiting low cytotoxicity and excellent biocompatibility and biodegradability. Due to their unique properties and high similarity to living tissues (Ossipov et al., 2021; Zheng et al., 2021), hydrogels have gained increasing attention for various biomedical applications, such as wound dressing and tissue engineering (Rinker et al., 2014; Norouzi et al., 2016; Solana Muñoz et al., 2018; Ghavimi et al., 2019; Ning et al., 2019). However, their use in bone regeneration is often hampered due to a lack of mineralization. Thus, hydrogels are loaded with anti-osteoporosis adjuvants to form a delivery system that can effectively compensate for this deficiency. In recent years, the development of hydrogel-related materials to treat osteoporosis has garnered significant interest, and given the encouraging results obtained, for instance, a locally-applied treatment was found to inhibit peri-implant bone resorption, while enhancing peri-implant bone formation and implant stability (Fu et al., 2016; Kettenberger et al., 2017). In this review, we summarize the preclinical research literature on the local treatment of osteoporosis with hydrogel-based delivery systems developed in the last decade, including different hydrogels and adjuvants. Hence, we hope to provide a valid starting point for subsequent research and clinical application of hydrogel-based delivery systems for osteoporosis therapy.

## 2 Hydrogel carriers

**FIGURE 1:**
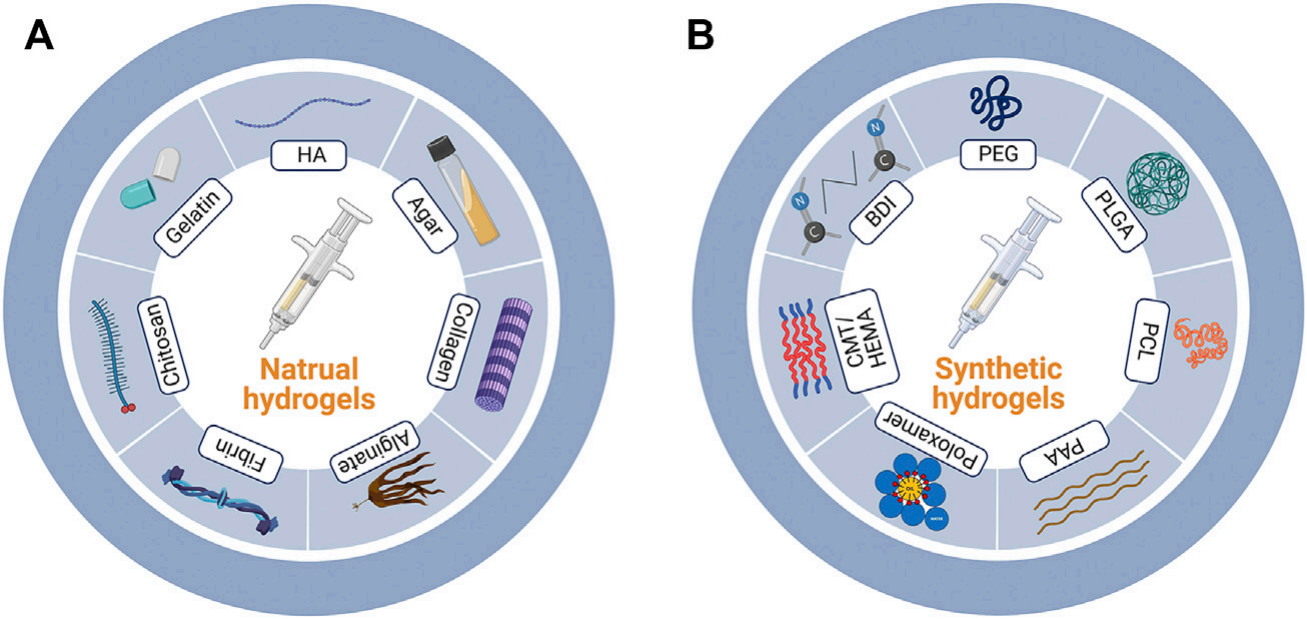
Nature-based and synthetic hydrogels. **(A)** Major components of nature-based and **(B)** synthetic hydrogels. HA, hyaluronic acid; PEG, polyethylene glycol; PLGA, poly (lactic-co-glycolic acid); PCL, polycaprolactone; PAA, polyacrylic acid; CMT/HEMA, carboxy methyl tamarind/hydroxyethyl methacrylate; BDI, butane-diisocyanate.

According to the types of the components, here in this review, we grouped the hydrogels into nature-based ones and synthetic ones (Figure 1). Besides, composite materials containing several types of hydrogels were also discussed. The detailed discussion is listed as follows.

### 2.1 Nature-based hydrogels

Nature-based hydrogels are derived from cross-linking components derived from nature. These are characterized with low cytotoxicity, excellent biocompatibility, and biodegradability (Table 1; Ito et al., 2012; Ito and Otsuka, 2013; Jiang et al., 2014; Ye et al., 2014; Lee et al., 2015; Cancian et al., 2016; Ma et al., 2016; Pelled et al., 2016; Kim et al., 2017; Kim et al., 2018; Nafee et al., 2018; Solana Muñoz et al., 2018; Ghavimi et al., 2019; Liang et al., 2019; Ning et al., 2019; Ossipov et al., 2021; van Houdt et al., 2021; Yilmaz et al., 2021). The commonly used materials include hyaluronic acid (HA), chitosan, collagen, gelatin, alginate, fibrin, agar and so on.

**TABLE 1:** Components and characteristics of nature-based hydrogels.

Hydrogels	Literatures	Characteristics
HA, Ca^2+^	Ossipov et al. (2021), van Houdt et al. (2021)	self-healing, two drugs released independently in response to acidic and thiol-containing microenvironments
HA, β-TCP	Lee et al. (2015)	porous β-TCP microspheres
Chitosan, β-glycerophosphate	Nafee et al. (2018)	Thermoreversibility
Chitosan, hydroxyapatite, carbon nanotubes	Cancian et al. (2016)	thermosensitive, higher resistance to compression, controlled release of protein drugs
Chitosan, cellulose nanocrystal	Ghavimi et al. (2019)	mechanical strength similar to vertebral bone, osteoinductivity
Collagen, hydroxyapatite, genipin	Ma et al. (2016)	improved mechanical property, higher gel content, lower swelling ratio, and tunable degradation behaviors against collagenase
Collagen I	Jiang et al. (2014)	NA
Methacrylated gelatin	Ning et al. (2019)	prolonged drug release (>10 days)
Gelatin, HPA	Kim et al. (2018)	NA
Calcium alginate	Ye et al. (2014)	NA
Alginate, CaP	Ito et al. (2012)	slow-releasing drug reservoir, protective coating, pH buffer
	Ito and Otsuka, (2013)	
Silk fibroin	Liang et al. (2019)	NA
Fibrin	Kim et al. (2017)	commercially available
	Pelled et al. (2016)	
Agar, hydroxyapatite	Solana Muñoz et al. (2018)	screw augmentation effect
Silica-quince seed mucilage	Yilmaz et al. (2021)	osteogenesis

HA, hyaluronic acid; β-TCP, β-tricalcium phosphate; HPA, hydroxyphenyl propionic acid; CaP, calcium phosphate; NA, not available.

#### 2.1.1 Hyaluronic acid

HA is a natural mucopolysaccharide acidic polymer and a main component of the extracellular matrix. It is widely distributed on the market as a hydrogel carrier (Kettenberger et al., 2017) owing to its excellent biocompatibility, high amenability, and multifunctional properties (Qiu et al., 2021), which make it an optimal candidate for tissue engineering as well. Ossipov et al. (2021) reported an injectable and self-healing HA hydrogel that independently released two different drugs in response to acidic and thiol-containing microenvironments. Except for the hydrogel carrier itself, the binding sites available on each individual component must also be considered when designing hydrogels. For instance, the BP prodrug is conjugated to HA *via* a self-immolative disulfide linker, that is, stable in the blood plasma and cleavable in the cytoplasm; the resulting HA-linked BP ligands reversibly bind Ca^2+^ ions and form coordination hydrogels (Ossipov et al., 2021).

#### 2.1.2 Chitosan or chitin

Chitosan, a linear and semi-crystalline polysaccharide, is a direct derivative of chitin, which is the second most abundant natural polymer after cellulose (Friedman and Juneja, 2010; Gong et al., 2022). Chitosan has excellent biocompatibility, biodegradability, adsorption capacity, anti-bacterial properties, and thermosensitive properties (Cancian et al., 2016; Nafee et al., 2018). It can be easily produced through acylation, alkylation, and carboxylation reactions (Shariatinia, 2019). However, chitosan-based hydrogels often fail to meet the mechanical strength requirements of bone fillers. The poor mechanical strength of chitosan-based hydrogels can be compensated by incorporating several types of nanofillers, i.e., carbon nanotubes and cellulose nanocrystals, which is particularly important for treating managing osteoporotic vertebral compression fractures (Cancian et al., 2016; Ghavimi et al., 2019; Vitale et al., 2022).

#### 2.1.3 Collagen or gelatin

Gelatin is a type of protein obtained by the partial hydrolysis of collagen; both have similar homologies. Collagen has a rod-like triple-helical structure, which is partially separated and broken when it is partially hydrolyzed to make gelatin (Veis and Cohen, 1960). Collagen-based hydrogels formed under physiological conditions using genipin as a cross-linker exhibited markedly improved mechanical properties, higher gel content, lower swelling ratio, and tunable degradation behaviors against collagenase (Ma et al., 2016). In addition, Ning et al. (2019) reported that the methacrylated gelatin-based hydrogel prolonged the release of the adjuvant (>10 days).

#### 2.1.4 Alginate

Alginate, derived from algae, is a linear copolymer composed of beta-d-mannuronic acid and C-5-epimer alpha-l-guluronic acid. An alginate solution gels when exposed to Ca^2+^ and other bivalent cations as these cations strongly bind to the G residues of the alginate molecule. Therefore, the calcium alginate gel is considered a three-dimensional network of molecules with cross-links between the G residues of other long-chain molecules through the action of Ca^2+^ (Bjerkan et al., 2004). The drug-release kinetics from hydrogels are commonly controlled by the network properties and drug–network interactions (Ossipov et al., 2021). The alginate gel sometimes degrades too rapidly under acidic conditions, such as the area around the osteoporotic bones. Thus, it has been reported that adding amorphous CaP powders positively affected dissociation rate (Ito et al., 2012; Ito and Otsuka, 2013), owing to the pH buffering mechanism inside the gel, thereby allowing for a controlled drug release.

### 2.2 Synthetic hydrogels

Synthetic hydrogels are covalently cross-linked with synthetic materials (Sanyasi et al., 2014; Fu et al., 2016; Tan et al., 2016; Neuerburg et al., 2019; Li et al., 2020; Zhao et al., 2020; Wang X. et al., 2021; Chen et al., 2021) (Table 2). Apart from the excellent mechanical and swelling properties, some of them allow for a controlled drug release (Zheng et al., 2021).

**TABLE 2:** Components and characteristics of synthetic hydrogels.

Hydrogels	Literatures	Characteristics
Poloxamer 407	Chen et al. (2021)	commercially available, thermosensitive
	Wang et al. (2021)	
	Tan et al. (2016)	
	Fu et al. (2016)	
BDI	Neuerburg et al. (2019)	commercially available, thermosensitive
Tetra-PEG	Li et al. (2020)	biocompatible, rapid gel formation, excellent injectability
PAA, nano-hydroxyapatite, sodium carbonate	Zhao et al. (2020)	initial morphology and mechanical properties maintained under physiological conditions, good primary stability, biocompatibility, bioactivity, and osteoconductivity
CMT/HEMA	Sanyasi et al. (2014)	effective adhesion, growth and further clustering of bone precursor cells, without any cytotoxicity

BDI, butane-diisocyanate; PEG, polyethylene glycol; PAA, polyacrylic acid; CMT/HEMA, carboxy methyl tamarind/hydroxyethyl methacrylate.

#### 2.2.1 Poloxamer 407 and butane-diisocyanate

Some commercially available products, such as Poloxamer 407 and butane-diisocyanate, can be directly used as hydrogels to carry adjuvants and are mainly used to study the function of targeted drugs (Neuerburg et al., 2019; Chen et al., 2021). Other commonly used synthetic materials used to produce hydrogels are reported below.

#### 2.2.2 Polyethylene glycol

PEG, also known as a macrogol, is a type of nontoxic and water-soluble polymer with unique hydrophilicity and electrical neutrality. It consists of chemically active hydroxyl groups at both ends, thereby promoting the conjugation with other functional groups (Alper and Pashankar, 2013; Shi et al., 2021). It can rapidly form biocompatible gels and is easily injectable; therefore, it is considered an ideal hydrogel carrier (Li et al., 2020).

#### 2.2.3 Poly lactic-co-glycolic acid

PLGA is a biodegradable polymeric compound formed by polymerizing lactic acid and glycolic acid, which are by-products of human metabolic pathways. Therefore, it is nontoxic, except in those individuals suffering from lactose deficiency. The co-polymerization between PLGA and the carried drug may prolong the release time; this confirms that the release of the drugs from the carrier depends on the degradation of the hydrogel and the decomposition of the drug complex (Liu et al., 2017; Peng et al., 2017). Additionally, PLGA can co-polymerize with PEG to form new polymers for hydrogel-based delivery systems (Liu et al., 2017).

#### 2.2.4 CHAp-polyacrylic acid

CHAp-PAA is the term used to refer to a supramolecular hydrogel composed of nano-hydroxyapatite, sodium carbonate, and polyacrylic acid (PAA) (Zhao et al., 2020); owing to the high mineralization, such hydrogel can be used as a scaffold to treat bone defects in osteoporotic individuals. Because of the biomineral composition, the hydrogel can mimic the chemical composition and structural characteristics of natural bones, while achieving mechanical stability, biocompatibility, and osteogenesis without delivering any additional therapeutic agents or stem cells (Zhao et al., 2020).

#### 2.2.5 Carboxy methyl tamarind/hydroxyethyl methacrylate

CMT/HEMA (ratio of 1:10) is a hydrogel with a surface that promotes the adhesion of bone precursor cells and efficient growth of bone tissues (Sanyasi et al., 2014). It is highly compatible with bone cells (RAW264.7) and sensitive to neuronal (Neuro2a) and human umbilical vein endothelial (HUVEC) cells (Sanyasi et al., 2014).

### 2.3 Composite hydrogels

While nature-based hydrogels are unable to withstand the pressure at the site of the bone injury because of their poor mechanical properties, their synthetic counterparts generally exhibit poor biocompatibility, lack interactions with targeted cells, and tend to cause adverse reactions in the body (Zheng et al., 2021). Composite hydrogels that combine the advantages of natural and synthetic hydrogels have been proposed to overcome these individual drawbacks (Utech and Boccaccini, 2016; Gačanin et al., 2017; Kettenberger et al., 2017; Kim et al., 2018; Segredo-Morales et al., 2018; García-García et al., 2019; Ghavimi et al., 2019; Akbari et al., 2020; Bai et al., 2020; García-García et al., 2020; Gilarska et al., 2021; Yoon et al., 2021); some of these composite hydrogels with their respective characteristics are reported in Table 3. However, given the treatment required to treat osteoporosis, a prolonged drug release time was the main aim while designing the target composite hydrogel, as discussed below.

**TABLE 3:** Components and characteristics of composite hydrogels.

Hydrogels	Literatures	Characteristics
Hydroxypropyl chitin, HA	Yu et al. (2020b)	thermosensitive, tunable biodegradable property, long-term sustained drug release (>28 days) with considerable structure stability, compatibility, osteoconductive potential
Hydroxypropyl chitin, alginate, Ca^2+^	Yu et al. (2020a)	thermosensitive, long-term sustained drug release (>28 days) with conformation stability, biocompatible, osteoconductive potential
Chitosan, glycerophosphate, gelatin	Akbari et al. (2020)	High gel strength, slow drug release rate, promotive effect on differentiation of osteoblasts
Biopolymeric collagen/chitosan/hyaluronic acid matrix, amine group-functionalized silica particles decorated with apatite, genipin	Gilarska et al. (2021)	lack systemic toxicity, particularly useful for the repair of small osteoporotic bone defects
HA, PVA, hydroxyapatite	Kettenberger et al. (2017)	commercially available
Gellan gum, PLGA	Posadowska et al. (2015)	low release rate
N-chitosan, ADH, HA-ALD	Bai et al. (2020)	self-healable, injectable, and biodegradable, functions and structures retained after external damage
Alginate, PLGA	García-García et al. (2020)	injectable, lower porosity, water absorption capacity and bone repair compared with its solid sponge state
Alginate, PLGA-PEG-PLGA, Ca^2+^	Liu et al. (2017)	thermosensitive, controlled drug release
Chitosan, collagen, 2-hidroxipropil γ-ciclodextrin, nanoparticles of hydroxyapatite, PLGA	García-García et al. (2019)	controlled drug release
Human serum albumin, ssDNA, PEG	Gačanin et al. (2017)	biocompatible, biodegradability, rapid gelation under physiological conditions, self-healing, spatiotemporally controlled release of active proteins
Gelatin, PNIPAM, PDMS	Yoon et al. (2021)	highly interconnected, dense channel networks
PF127, T1307, CD, PLGA, PLA	Segredo-Morales et al. (2018)	thermoresponsive
mPEG-PLGA	Peng et al. (2017)	extended drug release, biocompatibility
PLGA, PCL, capryol 90	Hosny and Rizg, (2018)	higher bioavailability, extended drug release (>3 months)
HA, PF127	Akbari et al. (2020)	commercially available

HA, hyaluronic acid; PVA, polyvinyl alcohol; PLGA, poly (lactic-co-glycolic acid); N-chitosan, N-carboxyethyl chitosan; ADH, adipic acid dihydrazide; HA-ALD, hyaluronic acid-aldehyde; PEG, polyethylene glycol; PNIPAM, poly (N-isopropylacrylamide); PDMS, polydimethylsiloxane; PF127, Pluronic F127; T1307, Tetronic 1307; CD, α-cyclodextrin; PLA, poly lactic acid; PCL, polycaprolactone.

The combination of natural hydrogels showed interesting results, as reported in several studies (Akbari et al., 2020; Yu et al., 2020a; Yu et al., 2020b) investigating the combination of chitin and chitosan for this scope; the resulting composite materials showed a good adsorption capacity coupled with a longer drug release time. Yu et al. combined hydroxypropyl chitin and HA or alginate, which exhibited a prolonged (28 days) and controlled drug release and considerable structure stability, and the studies showed a higher ALP activity, calcium expression and extracellular calcium deposition without inflammation and immune responses, indicating its potential for osteoconductive applications (Yu et al., 2020a; Yu et al., 2020b).

For composite hydrogels formed by the combination of natural and synthetic, or synthetic and synthetic components, PLGA is often added to prolong the drug release time (Posadowska et al., 2015; Liu et al., 2017; Peng et al., 2017; Hosny and Rizg, 2018; García-García et al., 2019). PLGA is commonly used in such composite systems to form microsphere structures. In the study by García-García et al. (2019), the hydrogel core of a sandwich-like system composed of the chitosan-collagen complex, 2-hidroxipropil-ciclodextrina and hydroxyapatite nanoparticles, and the addition of PLGA-based microspheres controlled the release of the adjuvants. The system placed in the defect easily adapted to the shape; after 12 weeks, approximately 50% of the defect was refilled with new tissue. Hosny and Rizg, (2018) adopted a Box–Behnken experimental design while using the Statgraphics^®^ software to develop an *in situ* hydrogel. The composite material, composed of PLGA, PCL, and the lipid surfactant capryol^®^90, exhibited a high bioavailability and extended drug release (>3 months), which aided in minimizing the side effects of several anti-osteoporosis drugs (Hosny and Rizg, 2018).

Several other composite hydrogels have been developed based on different application requirements. The circulatory system is the major route used to deliver drugs. Thus, a highly interconnected and dense channel network can be achieved by combining gelatin, poly (N-isopropylacrylamide), and polydimethylsiloxane. This composite material overcame the 200 μm diffusion limit of any 3D hydrogel (Yoon et al., 2021) and aided the recovery of the endocrine function. Moreover, it led to a full endometrium regeneration in the osteoporotic models, while effectively suppressing the side effects observed with the synthetic hormone treatment and preventing the representative aftereffects of menopause (Yoon et al., 2021). Lately, the self-healing capacity reported for some types of hydrogels has also received attention (Gačanin et al., 2017; Bai et al., 2020). Adipic acid dihydrazide (ADH), which is also a cross-linking agent promoting the formation of relatively stable hydrazone links from aldehydes (Bystrický et al., 1999) is one such example. It is cross-linked with N-carboxyethyl chitosan (N-chitosan) and hyaluronic acid-aldehyde (HA-ALD) *in situ* to form an injectable and self-healing supramolecular hydrogel. This composite hydrogel exhibited a remarkable self-healing capacity and retained its structural integrity after it was subjected to external damage (Bai et al., 2020).

## 3 Loaded anti-osteoporosis adjuvants

**FIGURE 2:**
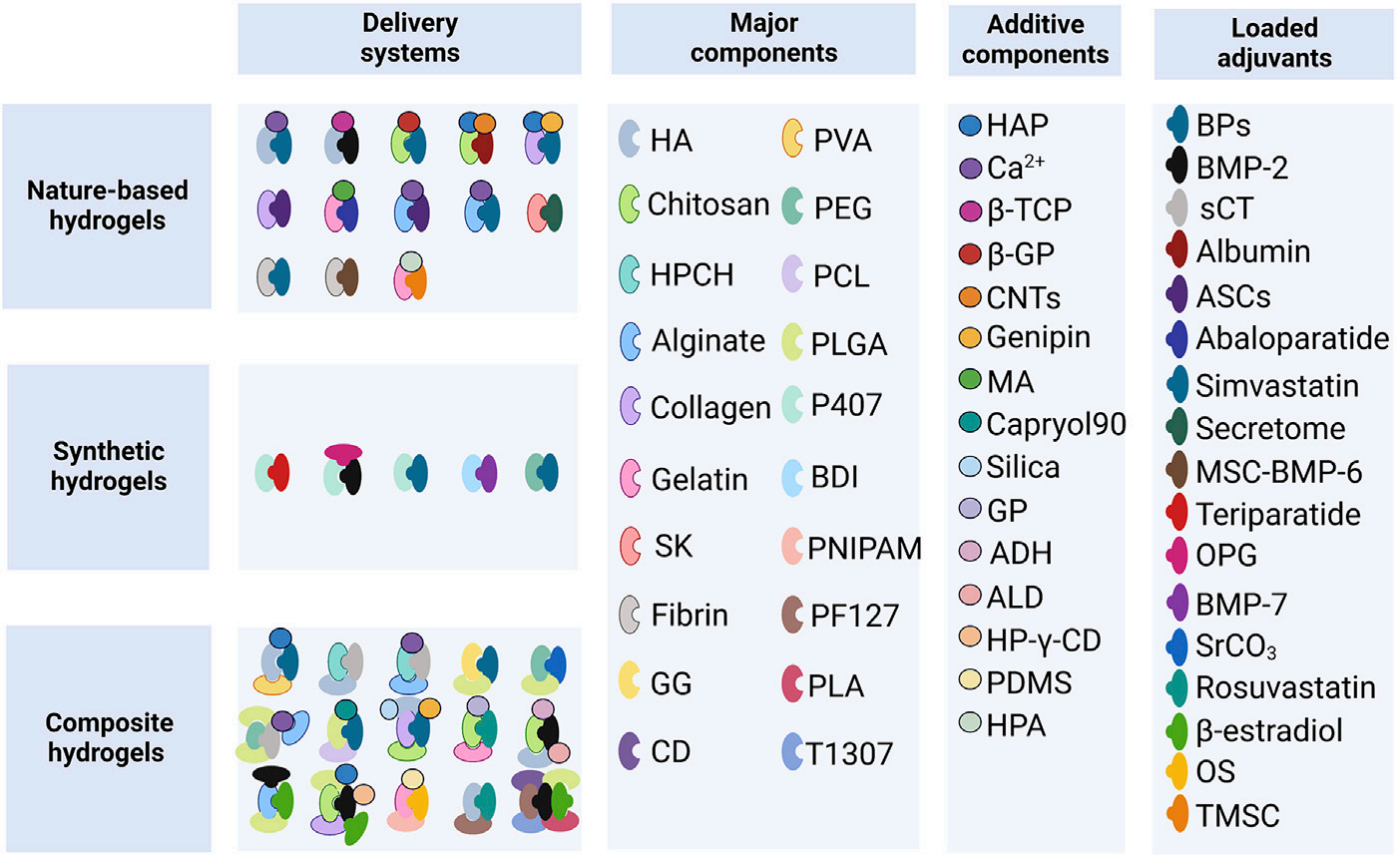
Hydrogel-based delivery systems. HA, hyaluronic acid; PVA, polyvinyl alcohol; HPCH, hydroxypropyl chitin; PEG, polyethylene glycol; PCL, polycaprolactone; SK, silk fibroin; GG, gellan gum; PLGA, poly (lactic-co-glycolic acid); P407, Poloxamer 407; BDI, butane-diisocyanate; PNIPAM, poly (N-isopropylacrylamide); PF127, Pluronic F127; T1307, Tetronic 1307; CD, α-cyclodextrin; PLA, poly-lactic acid; HAP, hydroxyapatite; β-TCP, β-tricalcium phosphate; β-GP, β-glycerophosphate; CNTs, carbon nanotubes; MA, methacrylic anhydride; GP, glycerophosphate; ADH, adipic acid dihydrazide; ALD, aldehyde; HP-γ-CD, 2-hidroxipropil γ-cyclodextrin; PDMS, polydimethylsiloxane; HPA, hydroxyphenyl propionic acid; BPs, bisphosphonates; BMP-2, bone morphogenetic protein-2; sCT, salmon calcitonin; ASCs, adipose-derived stem cells; MSC-BMP6, porcine mesenchymal stem cells overexpressing the BMP6 gene; OPG, osteoprotegerin; BMP-7, bone morphogenetic protein-7; OS, ovarian spheroids; TMSC, tonsil-derived mesenchymal stem cells.

The human skeleton is composed of the cortical and cancellous bone (the main part of the vertebrae). Bone remodeling is a process in which osteoclastic bone resorption and osteoblastic bone formation are regulated to achieve a dynamic balance in young adults (Hattner et al., 1965). The process of aging is associated with a negative remodeling balance, resulting in bone mass loss and disruption of the bone microarchitecture (Compston et al., 2019). The genetic factors account for 50%–85% of the normal variance in bone mass. The general signal pathways in osteoporosis include the receptor activator of nuclear factor κB (RANK), RANKL, osteoprotegerin (OPG), bone morphogenic protein (BMP), and Wingless-related integration site (Wnt) (Rachner et al., 2011). As discussed below, the loaded adjuvants mainly inhibit osteoclast formation or promote osteoblasts formation; several systems composed of the hydrogel-based carrier and adjuvant have been investigated thus far (Figure 2).

### 3.1 Inhibition of osteoclastic bone resorption

#### 3.1.1 Osteoclast differentiation pathways and bone resorption function

To treat osteoporosis successfully, it is important to inhibit the formation, differentiation, and resorption functions of osteoclasts. Osteoclasts are a special type of terminally differentiated cell deriving from a family of mononuclear macrophages in the blood. They can be fused by their mononuclear progenitor cells in various ways to form a multinuclear giant cell, where the RANKL and macrophage colony-stimulating factor (M-CSF) play a crucial role (Rachner et al., 2011). Tumor necrosis factor superfamily 11 (TNFSF11), the gene encoding RANKL, is abundantly expressed by osteoblasts, bone marrow stromal cells, and T and B lymphocytes (Rachner et al., 2011). As a homotrimeric type II transmembrane protein, RANKL can be released from the cell membrane upon decomposition of several extracellular proteases, including disintegrin and metalloprotease (Nagy and Penninger, 2015). RANK and OPG are the two main receptors of RANKL; such receptors are also known as tumor necrosis factor receptor superfamily member 11A (TNFRSF11A) and TNFRSF11B, respectively (Nagy and Penninger, 2015).

**FIGURE 3:**
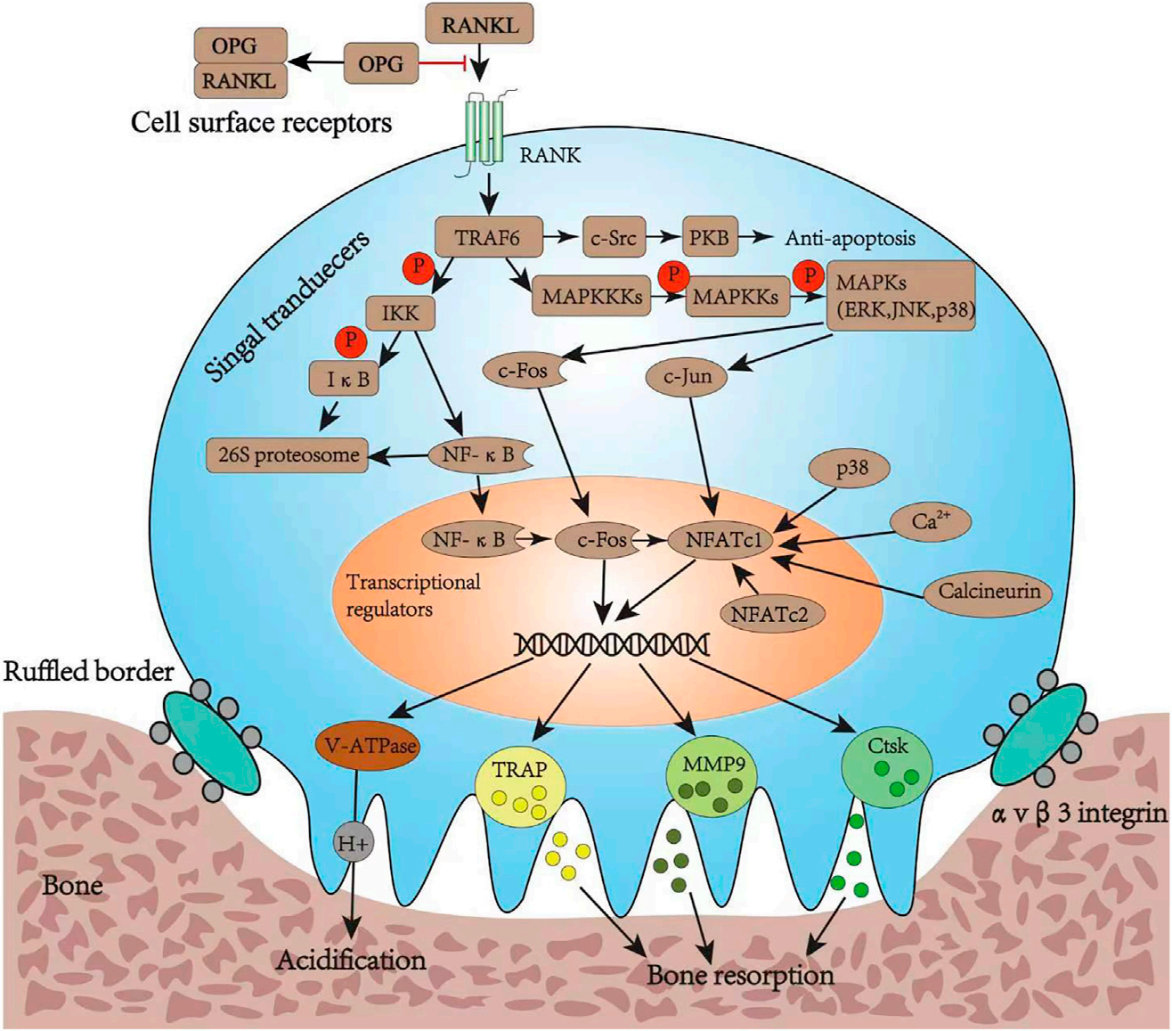
Osteoclast differentiation pathways and its bone resorption function. RANKL, ligand of receptor activator of nuclear factor κB; RANK, receptor activator of nuclear factor κB; OPG, osteoprotegerin; TRAF6, tumor necrosis factor receptor-associated factor 6; IKK, inhibitor of nuclear factor κB kinase; IκB, inhibitor of nuclear factor kappa B; NF-κB, nuclear factor-κB; PKB, protein kinase B; MAPKKK, mitogen-activated protein kinase; MAPKK, mitogen-activated protein kinase; MAPK, mitogen-activated protein kinase; ERK, extracellular regulated protein kinase; JNK, c-Jun N-terminal kinase; NFATc1, nuclear factor of activated T cell cytoplasmic 1; NFATc2, nuclear factor of activated T cell cytoplasmic 2; V-ATPase, vacuolar H (+) ATPase; TRAP, tartrate-resistant acid phosphatase; MMP9, matrix metalloproteinase 9; Ctsk, cathepsin K.

When secreted RANKL binds to the membrane-binding receptor RANK on the precursor of osteoclasts, it causes the RANK receptor to polymerize into a trimer that recruits several junction molecules, including tumor necrosis factor receptor-associated factor 6 (TRAF6) (Armstrong et al., 2002). The recruitment of TRAF6 leads to the activation of a variety of signaling pathway cascades, including inhibitor of nuclear factor κB kinase (IKK), mitogen-activated protein kinase (MAPK) family, and cellular Src kinase (c-Src), which enable osteoclasts to differentiate, survive, polarize, and have absorptive activity. The MAPK pathway is composed of three types of molecules: MAPK (including ERK, JNK, and p38), MAPK kinase (MAPKK or MEK), and MAPKK kinase (MAPKKK or MEKK) (Lee et al., 2018). The activation of MAPKs induces the nuclear translocation of c-Fos and c-Jun, while nuclear factor-κB (NF-κB), derived from the IKK pathway, up-regulates c-Fos in the nucleus upon nuclear translocation. The c-Src activates the anti-apoptotic program through protein kinase B. The nuclear factor of activated T-cells cytoplasmic 1 (NFATc1) is a critical transcription factor for osteoclast differentiation. Upon the initial activation through NF-κB and NFATc2, it is then upregulated by the action of c-Jun, p38, c-Fos, calcineurin, and calcium ions (Zhao et al., 2010). Finally, the up-regulated c-Fos and NFATc1 synergistically promote the expression of osteoclast-specific genes, such as tartrate-resistant acid phosphatase (TRAP), cathepsin K (CtsK), matrix metalloproteinase-9 (MMP-9), and vesicle-type ATPase (V-ATPase) V0 domain d2 subunit (Teitelbaum, 2000; Boyle et al., 2003; Nagy and Penninger, 2015). Bone degradation includes polarization, acidification, and protein breakdown; during these steps, the ruffled bone edge formed by osteoclasts plays an important role. In fact, it secretes protons (H^+^) into the bone resorption space through the V-ATPase and proteases such as TRAP, CtsK, and MMP-9; while the former degrade the bone minerals, the latter deteriorate the organic bone components (Georgess et al., 2014) (Figure 3).

#### 3.1.2 Adjuvants commonly used to inhibit osteoclasts

The binding between RANKL and RANK, and the following signaling cascade play an important role in osteoclast differentiation and survival. The inhibition of these pathways has become a feasible target for the systematic or local treatment of osteoporosis (Matsumoto and Endo, 2021). Targeting extracellular pathways, OPG-loaded composite hydrogels capable of controlling the release of OPG can inhibit the binding of RANKL and RANK; therefore, the osteoclastic activation is reduced, while promoting bone regrowth and osseointegration in osteoporotic defects (Wang X. et al., 2021). Alendronate, which is widely used to alleviate osteoporosis by inhibiting osteoclasts, is one of the most common drugs loaded on hydrogels (Posadowska et al., 2015; Nafee et al., 2018; Li et al., 2020; Gilarska et al., 2021; Jiang et al., 2022); such a complex would likely inhibit the rate-limiting step in the cholesterol biosynthesis pathway, essential for osteoclast function (Fisher et al., 1999). In addition, it was observed that the loading of zoledronate does not affect its action; in fact, the inhibition of the degradation of the mineralized hydrogel and the resorption of the peri-implant bone are effectively carried out by the loaded and unbound zoledronate (Kettenberger et al., 2017).

### 3.2 Promotion of osteogenesis

#### 3.2.1 Osteogenic differentiation pathways

During osteoporosis development, bone marrow mesenchymal stem cells (MSCs) promote the depletion of osteoblasts while increasing the amount of adipocytes, thereby resulting in a slower bone formation rate and improved marrow fat accumulation (Moerman et al., 2004; Li et al., 2015). The specific differentiation direction is precisely regulated by factors in the signaling pathways, transcription factors, and microRNAs. Among numerous studies of signaling pathways, the wingless and int-1 (Wnt) classes and BMP represent two critical signaling pathways (Hu et al., 2018).

**FIGURE 4:**
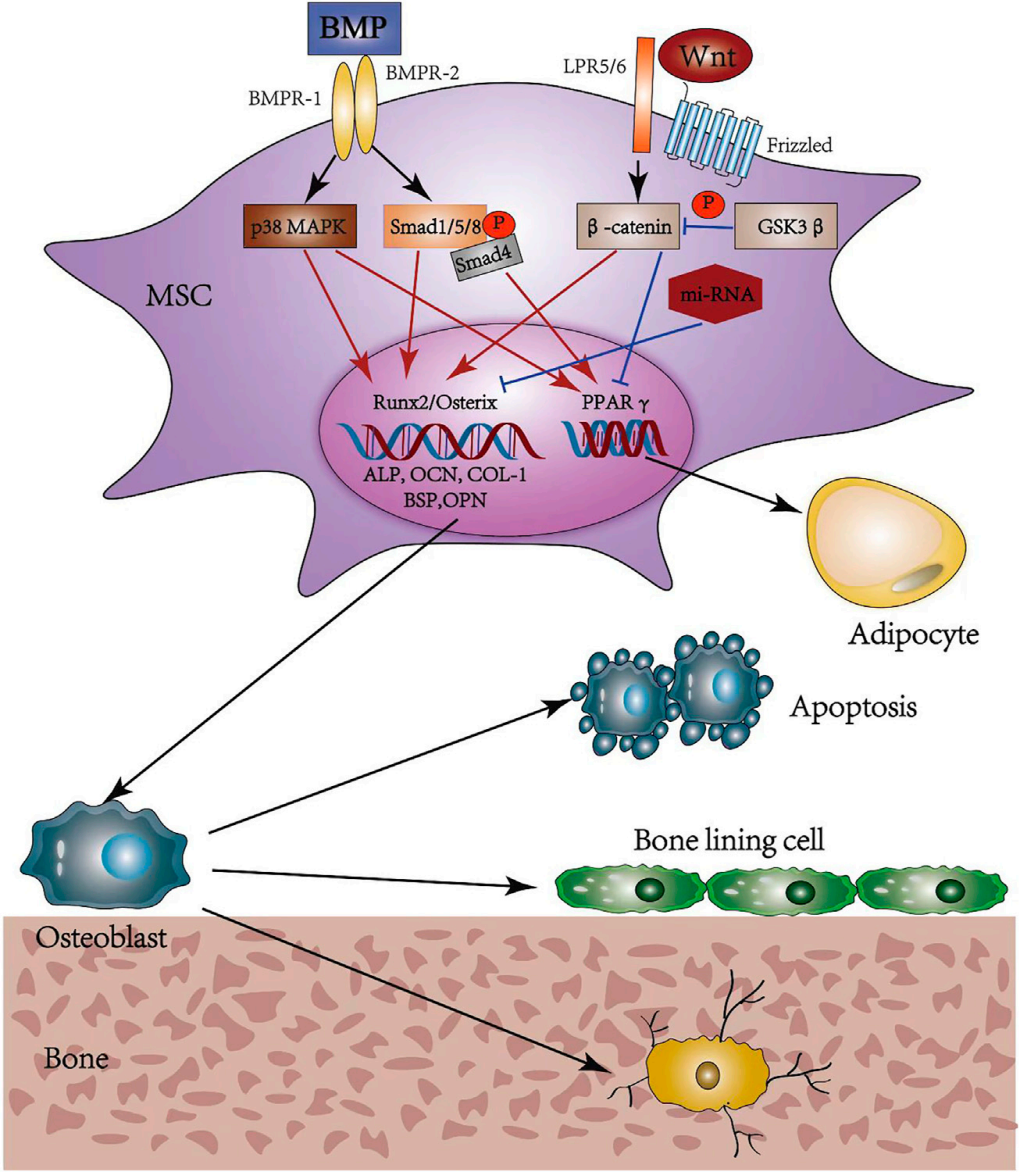
Osteogenic differentiation pathways and possible fates of osteoblasts. MSC, mesenchymal stem cell; BMP, bone morphogenic protein; BMPR, bone morphogenic protein receptor; MAPK, mitogen-activated protein kinase; Smad, small mothers against decapentaplegic; Wnt, wingless and int-1; LRP, low density lipoprotein receptor-related protein; GSK, glycogen synthase kinase; Runx2, runt-related transcription factor 2; ALP, alkaline phosphatase; OCN, osteocalcin; COL-1, collagen-1; BSP, bone sialoprotein; OPN, osteopontin; PPARγ, peroxisome proliferation-activated receptor γ.

The canonical Wnt signaling, also called Wnt/β-catenin, is essential for determining the fate of osteoblast cells. It binds a seven-transmembrane-spanning frizzled protein (Frz) receptor with the low-density lipoprotein receptor-related protein (LRP) 5/6 co-receptor to prevent the phosphorylation and degradation of β-catenin (Kim et al., 2013). Then, β-catenin translocates into the nucleus to promote osteogenesis while inhibiting adipogenesis, regulated by MSCs (Etheridge et al., 2004; Shen et al., 2011; Yuan et al., 2016). Moreover, this function may be achieved by inducing the expression of runt-related transcription factor-2 (runx-2) and osterix, and inhibiting peroxisome proliferation-activated receptor γ (PPARγ) (Bennett et al., 2005; Kang et al., 2007). BMP is the collective name for a series of transforming growth factor-β (TGF-β) family members and operates through either canonical or non-canonical BMP signaling (Chen et al., 2012) upon binding to bone morphogenetic protein receptor I (BMPR-I) and BMPR-II. The canonical BMP signaling induces phosphorylation of Smad1/5/8, which translocates into the nucleus upon the formation of complexes with Smad4; the non-canonical BMP signaling occurs mainly through the p38 MAPK pathway (Chen et al., 2012). Both signaling can regulate the target gene expression of runx-2, osterix, and PPARγ, showing dual roles in inducing osteogenic and adipogenic differentiation of MSCs (Kang et al., 2009). Several studies (Wang et al., 1993; Gori et al., 1999) indicate that a high BMP-2 concentration accelerates osteoblast differentiation, while adipocyte formation is promoted at low concentrations and in the presence of BMP-4 (Tang et al., 2004) (Figure 4).

#### 3.2.2 Adjuvants commonly used to promote osteogenesis

Since a high concentration of BMP-2 promotes osseointegration, it is one of the most common drugs loaded in hydrogel scaffolds (Lee et al., 2015; Segredo-Morales et al., 2018; García-García et al., 2019; Bai et al., 2020; García-García et al., 2020; Wang et al., 2021b; van Houdt et al., 2021). BMP-7 or BMP-6 loaded hydrogels have been used in local treatment, are potent stimulators of osteogenesis, and can reduce the risk of further osteoporosis-associated secondary fractures (Pelled et al., 2016; Neuerburg et al., 2019). Alternatively, Rosuvastatin is a popular drug that promotes osteogenic differentiation of MSCs in the model of osteoporosis by the Wnt/β-catenin signaling (Wang et al., 2019; Akbari et al., 2020). Simvastatin can also promote osteogenesis, and the underlying mechanism appears to involve a higher expression of BMP-2 (Ito et al., 2012; Fu et al., 2016; Tan et al., 2016; Zhu et al., 2021). In addition, carrying Si or Sr ions has been shown to promote osteogenic differentiation, with the former being controlled by upregulating the expression of the osteogenesis-related genes (Peng et al., 2017; Yilmaz et al., 2021). Hydrogels can also be directly loaded with MSCs, showing potential as a supplement or alternative to the current therapies proposed (Kim et al., 2018; Chang et al., 2019). Adipose-derived stem cells are also able to promote osteogenesis and inhibit adipogenesis of osteoporotic MSCs through activation of the BMP-2/BMP receptor-type IB signal pathway in the local delivery system (Jiang et al., 2014; Ye et al., 2014). Interestingly, it was observed that the treatment of drug-loaded hydrogels by extracorporeal shockwaves can promote bone formation by upregulating the alkaline phosphatase activity, mineralization, and expression of runx-2, type-I collagen, osteocalcin, and osteopontin (Chen et al., 2021).

### 3.3 Hormone analogs and osteo immunomodulators

The use of hormones and their analogs often play an important role in osteoporosis treatment, triggering complex physiological mechanisms, which have effects on osteoclasts and osteoblasts. Abaloparatide, as an analog of the human recombinant parathyroid hormone-related protein (PTHrp) that selectively binds to the RG conformation of the parathyroid hormone type one receptor, may represent a successful option for postmenopausal women affected by osteoporosis (Hattersley et al., 2016; Miller et al., 2016). Its role as a drug in hydrogel scaffold has also been proven (Ning et al., 2019). The hydrogel system containing calcitonin effectively reduced serum calcium levels, while promoting the reconstruction of bone trabecula (Liu et al., 2017; Yu et al., 2020b). Estrogens have also been tested for drug delivery in hydrogel carriers; for instance, 17β-estradiol is often locally delivered along with BMP (Segredo-Morales et al., 2018; García-García et al., 2019). In addition, hormone autocrination by vascularized hydrogel delivery of ovary spheroids (VHOS) to treat ovarian dysfunctions is successively conducted. The VHOS implantation effectively suppresses the side effects usually observed with synthetic hormone treatment, such as tissue overgrowth, hyperplasia, cancer progression, and deep vein thrombosis (Yoon et al., 2021). Moreover, recent studies have emphasized the use of immune cells in bone regeneration, giving rise to a new research field termed “osteoimmunology” (Liu et al., 2018; Fan et al., 2021). Among various innate immune cells, macrophages are one of the most vital effectors; as an example, they are the earliest cells approaching the implant area upon surgery. In the study of Jin et al. (2019) biomimetic hierarchical intrafibrillarly mineralized collagen loading IL-4 potently induced osteogenesis by promoting CD68^+^CD163^+^ M2 macrophage polarization in response to the critical-sized bone defects. In terms of other immune cells, hydroxyapatite nanorods with different aspect ratios could regulate osteogenesis through the modulation of T cells and IL-22 during bone regeneration (Yu et al., 2022).

## 4 Perspective and conclusion

As osteoporosis is a systemic disease, its local treatment has often been underestimated in the past; however, a hydrogel-adjuvant delivery system might lead to significant advantages in some specific cases, including local bone augment in surgery to prevent screw loosening or accelerating local bone healing after fracture. The inherent advantages of using natural hydrogels, i.e., good biocompatibility, cannot offset their lacking in mechanical properties; therefore, the modification of these natural components or the addition of synthetic ones is usually inevitable. The resulting composite hydrogel would ideally have excellent mechanical and swelling properties, controlled degradation and release rate, high drug-loading capacity, low cytotoxicity, and high biocompatibility. Regarding the loaded adjuvants, they should effectively promote osteoblasts while inhibiting the formation of osteoclasts. Despite the tremendous progress made in the field of tissue engineering over the past several decades, the passage from basic research to clinical application remains a critical challenge. The detection of the ideal hydrogel-adjuvant system has the potential to ease such a transition, but it requires the joint efforts of clinicians and researchers.
